# Towards More Accurate Determination of the Thermoelectric Properties of Bi_2_Se_3_ Epifilms by Suspension via Nanomachining Techniques

**DOI:** 10.3390/s22208042

**Published:** 2022-10-21

**Authors:** Donguk Kim, Chanuk Yang, Yun Daniel Park

**Affiliations:** 1 Department of Physics & Astronomy, Seoul National University, Seoul 08858, Korea; 2 Department of Physics, Research Institute of Physics and Chemistry, Jeonbuk National University, Jeonju 54896, Korea; 3 Institute of Applied Physics, Seoul National University, Seoul 08858, Korea

**Keywords:** Bi_2_Se_3_, MBE, thermoelectric, nanostructuring, 3*ω* method

## Abstract

We report on the characterization of the thermoelectric properties of Bi_2_Se_3_ epifilms. MBE-grown Bi_2_Se_3_ films on GaAs (111) A are nanomachined with integrated Pt elements serving as local joule heaters, thermometers, and voltage probes. We suspended a 4 µm × 120 µm Bi_2_Se_3_ by nanomachining techniques. Specifically, we selectively etched GaAs buffer/substrate layers by citric acid solution followed by a critical point drying method. We found that the self-heating 3*ω* method is an appropriate technique for the accurate measurement of the thermal conductivity of suspended Bi_2_Se_3_. The measured thermoelectric properties of 200 nm thick Bi_2_Se_3_ at room temperature were κ=1.95 W/m K, S=−102.8 μV/K, *σ* = 75,581 S/m and the figure of merit was ZT=0.12. The study introduces a method to measure thermal conductivity accurately by suspending thin films.

## 1. Introduction

Thermoelectric (TE) devices that create a voltage by heat waste have been under development for a few decades [1,2,3]. However, their applications are limited to a few industries due to their low efficiency in energy conversion [4,5]. The efficiency of TE devices is determined by the dimensionless figure of merit defined by the Seebeck coefficient (*S*), electrical conductivity (*σ*), thermal conductivity (*κ*) and the temperature (*T*). A high Seebeck coefficient, high electrical conductivity, and low thermal conductivity are required to maximize the figure of merit [6]. However, the interdependence of the Seebeck coefficient, electrical conductivity, and the electronic contribution to the thermal conductivity makes the optimization of ZT difficult. The electrical conductivity and electrical contribution to thermal conductivity are linked to each other by the Wiedemann–Franz law. According to the simple theory for nearly free electrons, the Seebeck coefficient of metals or degenerate semiconductors [7] decreases when the carrier concentration (*n*) increases. However, when the carrier concentration (*n*) increases, the electrical conductivity (*σ*) and electronic thermal conductivity ($\kappa_{e}$) increase [8].

To overcome these correlations between the quantities, one needs to maximize the power factor ($S^{2}\sigma$) by optimizing the carrier concentrations (10^19^~10^21^ carriers per cm^3^) [8] and/or minimize the lattice thermal conductivity ($\kappa_{l}$) by increasing phonon scattering. The introduction of point defects and doping effectively controls the carrier concentration and the power factor [9,10,11]. Nanostructuring, alloying, and the application of grain size and strain are used to enhance the phonon scattering and reduce the lattice thermal conductivity [12,13,14,15]. Because the mean free path (mfp) of electrons is much shorter than that of phonons in heavily doped semiconductors, nano-structuring effectively reduces the lattice contribution to thermal conductivity while preserving the electrical conductivity [12,16]. Recently, such material systems as phonon-glass electron-crystal, where phonon transport is confined in plane, have advanced thermoelectric research [17,18,19].

Despite the progress in nanostructuring reported for nanowires, superlattices, and nanocomposites in thermoelectric research of nano-films, there have been difficulties associated with heat diffusion to the substrate [20,21,22,23,24]. We present the self-heating 3ω method to measure the fundamental thermal properties by suspending the film [25,26,27]. Consider a suspended rod-like metal for thermal conductivity measurements [28]. An electrical current of the form $I_{0}\sin\omega t$ is sourced from outside probes. This results in Joule heating of the form $0.5I_{0}^{2}R\;\left(1-\cos2\omega t\right)$, where *R* is the resistance of the metal. This periodic Joule heating results in temperature oscillation at a frequency of 2ω, which causes the resistance of the metal to oscillate at 2ω. This leads to a voltage fluctuation at 3ω across the rod due to the injected electrical current at ω and the oscillation of the resistance at 2ω. The amplitude of the voltage at 3ω is determined by the thermal properties and described by the 1D heat diffusion equation. With proper boundary conditions and an initial condition, an explicit solution for the 1D heat-conduction equation at the low frequency limit can be found and the thermal conductivity (κ) can be measured from the excitation current (I), electrical resistance (R) and its differential respect to temperature ($R^{\prime }=dR/dT$), geometrical length (L) and cross-section area (A), and measured $V_{3\omega }$ signal. [29] This solution is obtained from the assumption that the joule’s heat fully dissipates in the rod and not in its surroundings, particularly the underlying substrate. A freely suspended structure best meets these important criteria. The 3ω methods can be applied to materials with a linear Ohmic current-voltage behavior.

The best TE materials typically degenerate semiconductors that have high carrier mobilities and a low electronic contribution to thermal conductivity [14]. Bi_2_Se_3_ is a semiconductor with a narrow bandgap of about 0.3 eV, which meets the conditions for a large Seebeck coefficient [9]. Naturally occurring Se-vacancies that act as electron donors result in high electrical conductivity, thus Bi_2_Se_3_ is known as one of the best TE materials [8]. A recent theoretical prediction of a topologically protected surface states in Bi_2_Se_3_ has rekindled interest in the development of high-quality materials [30,31]. In this study, we epitaxially grew 200 nm thick Bi_2_Se_3_ films on a GaAs (111) A substrate by molecular beam epitaxy (MBE). Nanostructuring techniques enable the accurate measurement of the thermal conductivity by freely suspending the Bi_2_Se_3_ from the GaAs substrate. Additionally, nanostructuring effectively suppresses the lattice’s contribution to thermal conductivity due to the low dimension [9].

## 2. Materials and Methods

**Figure 1:**
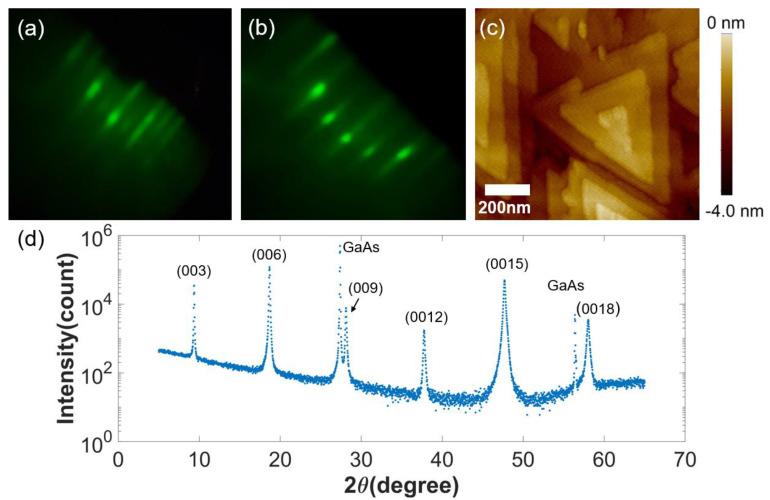
RHEED patterns during epitaxial growth of (**a**) the GaAs buffer layer and (**b**) Bi_2_Se_3_, (**c**) 1 μm × 1 μm AFM scan image, (**d**) high-resolution X-ray diffraction of Bi_2_Se_3_.

In this study, Bi_2_Se_3_ epifilms were grown on a semi-insulating GaAs (111) A substrate using effusion cells charged with Bi (99.9999%), Se (99.999+%), Ga (99.999%), and As (99.99999+%). In the growth chamber, after standard deoxidation, a substrate temperature of 700 °C, a 40 nm thick GaAs buffer layer was grown at a deposition rate of 1 nm/min under As-rich conditions. Then, 200 nm Bi_2_Se_3_ epifilms were grown at a substrate temperature of 300 °C with a growth rate of 2 nm/min under Se-rich conditions with the flux ratio (Φ_Se_/Φ_Bi_) of ~20 [32,33]. The growth rate and surface morphology were monitored by RHEED using oscillations and typical surface reconstructions (Figure 1a,b) [34,35]. Finally, a Se-capping layer was deposited below 150 °C to minimize the Se-vacancies. The resulting morphologies and crystal structures (Figure 1) are characterized by a commercially available non-contact mode AFM and from high resolution XRD measurements. Epifilms for this study all showed characteristic triangular pyramid morphologies with sizes and densities being dependent on the stoichiometry and other growth conditions. Thickness was measured by SEM and a profilometer.

**Figure 2:**
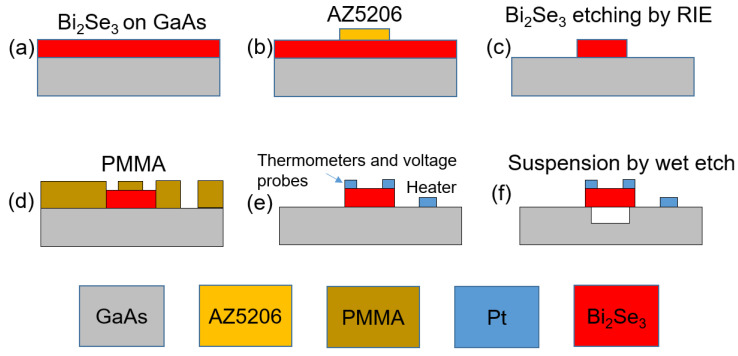
Schematic illustration of the nanomachining process used to freely suspend the Bi_2_Se_3_ epifilm. After mild thermal treatment to remove the Se capping layer (**a**), the Bi_2_Se_3_ structure was defined by standard photolithography methods (**b**) and formed by mild Ar plasma etching in RIE (**c**). Pt metallic thermal and electrical probes were patterned by standard e-beam lithography methods (**d**) and formed by lift-off after DC sputter deposition (**e**). Suspended structure was formed by selectively etching the underlying GaAs layer by a citric acid solution and drying it in a critical point dryer (**f**).

After epitaxy deposition, the samples were readied for nanofabrication by mild thermal treatment at 200 °C to remove the Se-capping layer. All nanopatterning was conducted by a modified commercially available SEM. The first alignment marks were realized after lift-off of DC sputter deposition of 20 nm thick Pt [36] after a negative resist process using an image reversal of AZ520X. Then, Bi_2_Se_3_ was patterned into a nanobeam structure (0.2 μm × 4 μm × 120 μm) by a similar image reversal process and mildly etched in a commercially available RIE in Ar plasma (0.015 mTorr and 150 W) with an etch rate of 100 nm/min. Then, necessary electrical probes and resistive thermometer in contact with a Bi_2_Se_3_ nanobeam as well as in-situ joule heaters situated on the GaAs substrate in proximity to the Bi_2_Se_3_ nanobeam were used again after the lift-off process of DC sputtered 200 nm Pt. To minimize substrate effects in thermal-transport measurements, the Bi_2_Se_3_ nanobeam was freely suspended by first patterning an etch window and then selectively etching the underlying GaAs using a mild citric acid solution (volumetric 6:1 ratio of citric acid and hydrogen peroxide) [37] at an etch rate of 100 nm/min. After suspending the Bi_2_Se_3_ nanobeam, the sample is dried in a commercially available critical point dryer (CPD). The final structure is shown in Figure 2f.

**Figure 3:**
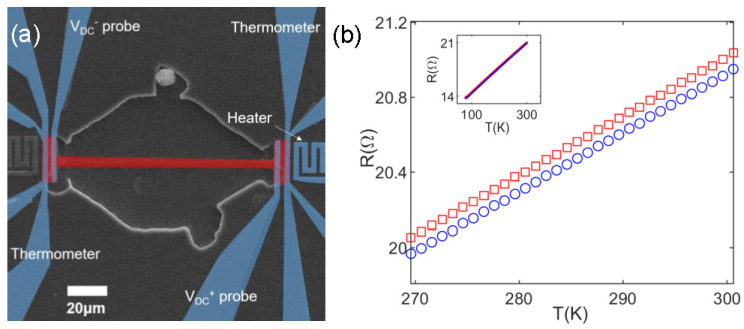
(**a**) False colored SEM image and schematic representation of the device for the Seebeck coefficient measurement. (**b**) Linear dependence of the resistance of Pt with the temperature (for the whole temperature range (inset)) when the Joule heater was ‘on’ (red square) and ‘off’ (blue circle).

After nanofabrication, electrical contacts were formed by Au wire-bonding. Electrical probes on Bi_2_Se_3_ were verified as being Ohmic from *I*–*V* measurements. Then, the fabricated Pt thermometer in proximity to the Bi_2_Se_3_ nanobeam was calibrated by measuring its resistivity while monitoring the sample temperature in a vacuum cryostat (<0.1 mTorr) using a commercially available temperature controller (LakeShore, Westerville, OH, USA, LS330). As expected, the Pt thermometer’s resistivity showed a linear dependence to the temperature range reported in this study (60–300 K) (Figure 3b). As the Pt thermometer was in physical contact with the Bi_2_Se_3_ nanobeam, the joule heater was placed in proximity to the nanobeam on the semi-insulating GaAs substrate. Accordingly, the thermometer and heater were electrically isolated.

Figure 3a schematically depicts the DC thermal-transport measurements (i.e., Seebeck effect). First, to measure the thermal gradient, the resistivities of the two thermometers were determined at substrate temperature. Then, a DC current was allowed to flow in one of the heaters and its magnitude was determined by a constant power condition of 100 mW while varying the substrate temperature. Then, the resistances of the two thermometers are measured using an ac-lock-in technique (17 Hz and 27 Hz with *I*_AC_ 5 μA) to determine the temperature gradient, which was found to be in the range 0.1 K/μm at 300–0.01 K/μm at 60 K. Considering that GaAs thermal conductivity greatly increases at lower temperatures and is an order of magnitude higher than that of Bi_2_Se_3_, this result (lower thermal gradient at lower temperature) indicates that the effect of the substrate (i.e., increase in thermal conductivity of the GaAs substrate at low temperature) cannot be easily ignored. After a time delay of 10 s after the heater is turned on to insure a thermal equilibrium, the DC voltage across the Bi_2_Se_3_ nanobeam was averaged and recorded after the probes, as the thermometers are disconnected by relay signals. The process was repeated from room temperature to 60 K while the substrate temperature was monitored and maintained.

**Figure 4:**
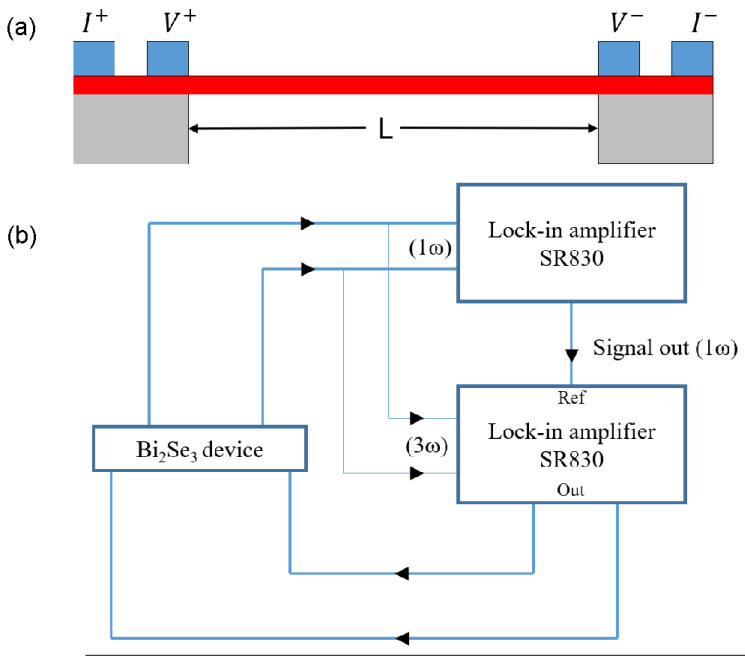
(**a**) Schematic cross-section of the self-heating 3*ω* thermal conductivity measurement, and (**b**) instrumentation used in this study.

**Figure 5:**
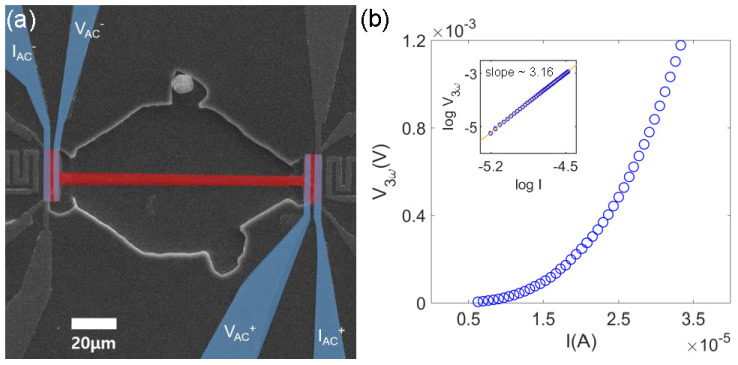
(**a**) SEM image of the device for measurement of the electrical conductivity and thermal conductivity. (**b**) The *I*-$V_{3\omega }$ measurement and log-log plot of *I*-$V_{3\omega }$ (inset) show the cubic relation at temperature 300 K.

Figure 4 schematically depicts AC thermal-transport measurements (i.e., thermal conductivity). We utilized two phased-locked ac lock-in amplifiers. To validate the 3*ω* method, we swept the ac current (*I_AC_*) at *f* = 13 Hz. One ac lock-in at *f* was used to measure the electrical ac conductivity of the Bi_2_Se_3_ nanobeam, while the other lock-in at 3*f* was recorded as $V_{3\omega }$. Figure 5b shows the expected cubic dependence of $V_{3\omega }$ to the injected current *I_AC_*. Similar to the above Seebeck effect measurements, the sample temperature was varied from room temperature to 60 K. From these measurements, we recorded the electrical conductivity *σ*, $V_{3\omega }$, and determined $R^{\prime }=\left[R\left(T_{1}\right)-R\left(T_{2}\right)\right]/\left[T_{1}-T_{2}\right]$.

## 3. Results

**Figure 6:**
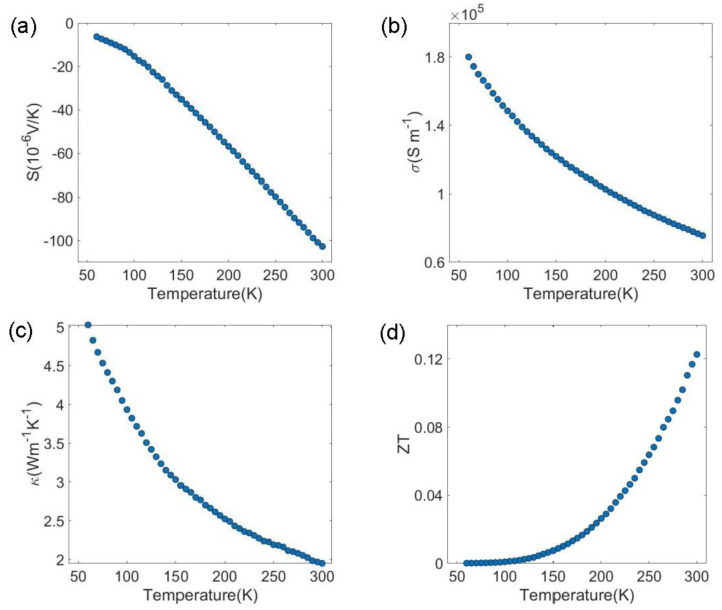
Thermoelectric properties of the 200 nm Bi_2_Se_3_ epifilm: (**a**) Seebeck coefficient temperature dependence; (**b**) electrical conductivity; (**c**) thermal conductivity; (**d**) calculated figure of merit (*ZT*).

From the Bi_2_Se_3_ epifilms, we successfully fashioned a versatile thermal-transport measurement platform in which the epifilm to be characterized is freely suspended to minimize thermal transport through the substrate. From in-situ RHEED monitoring during growth as well as post-growth morphologies characterized by non-contact mode AFM and high-resolution XRD, the resulting Bi_2_Se_3_ epifilms (Table 1) were found to compare favorably to those reported elsewhere [28,38,39,40]. Then, the resulting suspended Bi_2_Se_3_ with integrated electrical probes as thermometry in its proximity were then characterized in a cryogenic cryostat. First, the temperature dependence of the electrical conductivity of the as-grown Bi_2_Se_3_ epifilm and Bi_2_Se_3_ nanobeam is depicted in (Figure 6b). Characteristic temperature dependence indicate that the nanomachining processes had a minimal effect. At room temperature, for the 200 nm Bi_2_Se_3_ nanobeam, the electrical conductivity (*σ*) was 75,580 Ω^−1^·m^−1^. Again, this value is favorable compared with that of similarly sized bulk nanoribbon samples reported elsewhere [38].

Using the patterned thermometry elements near the Bi_2_Se_3_ nanobeam, Pt resistive thermometers in contact with Bi_2_Se_3_, and the Pt joule heater in proximity, we showed that a steady temperature gradient (ΔT ranging from 1 K to 10 K) can be maintained across the Bi_2_Se_3_ nanobeam for the temperature range presented here (60–300 K). Interestingly, for the equal heater power, higher temperature gradients were achieved at higher temperatures. This suggests that at lower temperatures, a significant amount of heat flows underneath the suspended Bi_2_Se_3_ nanobeam across the GaAs substrate. With a controllable temperature gradient and electrical probes to measure the electrical potential across the nanobeam, the Seebeck voltage can be directly measured. Figure 6a plots the Seebeck voltage for the 200 nm Bi_2_Se_3_ nanobeam structure. The sign of the voltage as well as the temperature dependence agree with the expected dominant n-type carriers from Se vacancies. Again, the Seebeck voltage of −102.8 μV/K for our 200 nm Bi_2_Se_3_ nanobeam compares favorably to values obtained from Bi_2_Se_3_ film grown by MBE [39], vapor-solid method [40] and Bi_2_Se_3_ nanoribbon synthesized by the vapor liquid solid method, which are also suspended from the substrate [38]. As the Seebeck voltage is expected to be dominated by thermal carrier diffusion, the fact that the absolute measured values of the Seebeck voltage for the nanobeam were slightly smaller than those of the nanoribbon possibly indicated a higher quality Bi_2_Se_3_ epifilm.

To ultimately quantize the thermoelectric figure of merit (*ZT*), we needed to accurately determine the thermal conductivity (*κ*) of the Bi_2_Se_3_ nanobeam. As mentioned above in the Seebeck voltage measurements, although the nanobeam is suspended from the substrate, we still found that a significant amount of heat was conducted through the substrate. Due to this uncertainty, we were not able to accurately determine *κ* by simply measuring the heating power and the temperature differently. Then, as mentioned in the introduction, we applied the 3*ω* method, specifically the self-heating method derived from the 1D heat diffusion equation [28]. As the heat was transferred directly to the nanobeam, the resulting measurements were robust with minimal substrate contributions.

Starting from the 1D heat diffusion model, thermal conductivity (*κ*) can be quantified by the self-heating 3*ω* method. An injected current of *ω* is used to “self-heat” the sample, and by trigonometric identity and joule heating, we can measure the *V_ω_* to determine the electrical conductivity (*σ*) and *V*_3*ω*_. Then, along with *σ* (*T*), *dR/dT*, and *V_ω_*, a temperature dependence of *κ* ($\approx \left[4RR^{\prime }LI^{3}\right]{\left[\pi^{4}AV_{3\omega }\right]}^{-1}$) can be seen as depicted in Figure 6c. At 300 K, the Bi_2_Se_3_ nanobeam *κ* was found to be ~1.95 Wm^−1^ K^−1^ (see Supplementary Materials). Similar to the nanoribbon sample [28,38], the measured value of *κ* and its temperature dependence compared favorably. The monotonic increase in *κ* with a decrease in temperature is consistent with metallic-like behavior found in Se-vacancy dominated Bi_2_Se_3_ materials. Along with the temperature dependent measurement of the Seebeck voltage, electrical conductance, and thermal conductance, the thermoelectric figure of merit (*ZT*) temperature dependence is plotted in Figure 6d. The uncertainty in our measurements is limited from thickness measurements, which we found from RHEED oscillations as well as stylus profilometer measurements and is typically within 5%. However, in determining the *ZT* values, the geometrical factors are cancelled out. With suppressed thermal conductivity from the reduced dimensionality of the nanobeam, we found the *ZT* values to be significantly larger than the reported bulk values at room temperature.

## 4. Summary

To more accurately determine the thermoelectric properties of Bi_2_Se_3_ epifilms, we utilized nanomachining techniques to freely suspend the structure. We also integrated thermometric and electrical probes. We applied the self-heating 3*ω* method to more accurately measure thermal conductivity. This method further minimizes heat transfer into the substrate and allows for more accurate determination as an ac measurement technique. From the characterization of the surface morphology and electrical measurements, we found our Bi_2_Se_3_ epifilms to be comparable to other reported epifilms. Electrical measurements conducted after nanofabrication processing showed minimal effects on the quality of the Bi_2_Se_3_. A reduction in the dimensionality of the epifilms resulted in a significant decrease in thermal conductivity compared to the bulk [41], with minimal decreases in the electrical conductivity and an increase in the Seebeck coefficient. Such changes in the thermal electric properties resulted in an increase in the figure of merit (*ZT*) compared with the bulk and reported studies, as in [41,42].

## Figures and Tables

**Table 1:** Summary of selected thermoelectric measurements of Bi_2_Se_3_.

Type of Bi_2_Se_3_	*κ* (W/mK)	*σ* (10^4^ S/m)	*S* (μV/K)	*ZT*
mechanically exfoliated Bi_2_Se_3_ [28]	~2.1	N/A	N/A	N/A
nanoribbon synthesized via VLS (S2) [38]	~1.7	~7	−120	0.17
30 nm epifilm [39]	N/A	~6	−100	N/A
77 nm vapor solid grown Bi_2_Se_3_ [40]	N/A	15.9	−99.9	N/A
200 nm epifilm (this report)	~1.9	~7.6	−103	0.12
bulk [41]	4	16	−70	0.05

## Data Availability

Not applicable.

## Supplementary Materials

The following supporting information can be downloaded at: https://www.mdpi.com/article/10.3390/s22208042/s1, Figure S1: Replot of Figure 5b in main text; Figure S2: Temperature dependence of 200 nm Bi_2_Se_3_ nanobeam.
