# Supplementary material for: Towards More Accurate Determination of the Thermoelectric Properties of Bi2Se3 Epifilms by Suspension via Nanomachining Techniques
Source: Sensors (Basel). 2022 Oct 21;22(20):8042. doi: 10.3390/s22208042 (PMC9609336; doi:10.3390/s22208042)
Supplement: Supplementary file 1 [file sensors-22-08042-s001.zip › sensors-1942031-supplementary.pdf]

Supplemental Material for

Towards more accurate determination of the thermoelectric properties of Bi_2Se_3 epifilms by suspension via nanomachining techniques: a detailed description of determining thermal conductivity (κ)

Donguk Kim, Chanuk Yang, and Yun Daniel Park

We utilize the self-heating 3ω method to accurately determine the thermal conductivity of the Bi_2Se_3 epifilm. An ac-electrical current ($I = I_o \sin \omega t$) is sourced across the nanomachined suspended Bi_2Se_3 nanobeam, which induce Joule heating ($P = 0.5 I_0^2 R(1 - cos2\omega t)$, where R is the electrical resistance of the nanobeam and a corresponding temperature and resistance fluctuation at 2ω. Then, the voltage across the nanobeam fluctuates at 3ω ($V = I_o \sin \omega t\, R_{2\omega}$). The amplitude of $V_{3\omega}$ can be determined from the 1D heat diffusion equation for metallic-like materials. Here, we assume that the nanomachined beam structure best allows us to approximate the 1D heat diffusion equation. An explicit solution can be found at a low frequency limit:

$$\kappa = \frac{4RR'L}{\pi^4 A} \frac{I^3}{V_{3\omega}}$$

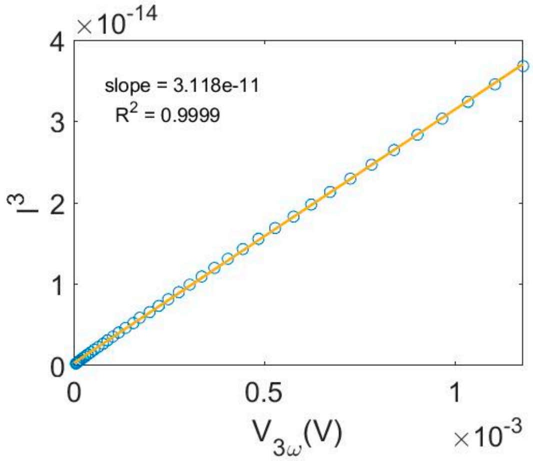

Figure S1 – Replot of figure 5(b) in main text.

The nanobeam structure also facilitates boundary and initial conditions. From temperature dependence of the electrical resistance as well as *I-V* measurements, we find the Bi_2Se_3 to be metallic-like with pure Ohmic behavior. To measure $V_{3\omega}$ and electrical resistance simultaneously, we had utilized two ac-lock-in amplifiers. We source a current by placing the nanobeam and a load resistor in series with a variable ac-voltage ($f = 13$ Hz). The lock-in ($f = 13$ Hz voltage) is recorded to determine the electrical resistance. A second lock-in ($f = 39$ Hz) records the $V_{3\omega}$.

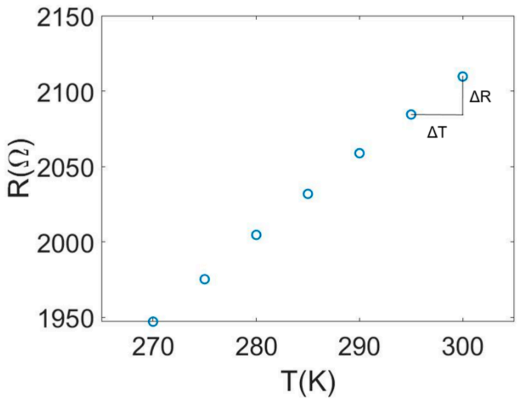

Figure S2 – Temperature dependence of 200 nm Bi_2Se_3 nanobeam.

We now detail the determination of thermal conductivity (κ) at 300 K. Figure S1, replots the figure 5(b) in the main text, a 200 nm Bi_2Se_3 nanobeam. The slope of the linear fit is related to ($\pi^4 A\kappa/4LRR'$). Figure S2, plots the temperature dependence of the same sample. The geometrical dimensions, length and width, of the beam is measured from SEM and thickness is measured by stylus profilometer and from RHEED oscillation calibrated growth rate and deposition time.

Then, the thermal conductivity can be calculated as

$$\kappa = \frac{4LRR'}{\pi^4 A} \frac{I^3}{V_{3\omega}} = \frac{4 \times 111\mu m \times 2110\Omega \times 5.23\Omega/\mathrm{K}}{\pi^4 \times 200nm \times 4.03\mu m} \times 3.12 \times 10^{-11} \mathrm{A}^3/\mathrm{V} = 1.95\mathrm{W/mK}$$
